# Valedictory Address

**Published:** 1878-05

**Authors:** R. N. Ely

**Affiliations:** Attorney General of Georgia


### VALEDICTORY ADDRESS TO THE GRADUATING CLASS IN ATLANTA MEDICAL COLLEGE, AT THE ANNUAL COMMENCEMENT, MARCH 1, 1878.

By R. N. Ely, Attorney General of Georgia.

Gentlemen—The part assigned me in these interesting exercises will occupy but a few minutes of your time. It is not to be expected that I should know any thing of the great science to which you are to be votaries, excepting in a very general way; but if I can drop a word of cheer and encouragement; if I can let fall some hint that may be of practical benefit to any of you, either in rendering less rugged the road over which you are to travel, or in enkindling in your hearts an increased enthusiasm for the work you have undertaken, then, indeed, shall I be glad that I am accorded the privilege of addressing you on this occasion.

You are now standing upon the threshold of active life. All of the past has been but a season of preparation for this hour. As neophytes, you are about to be enrolled in that grand universal army in which battles are to be fought, victories won, and defeats endured.

The business to which you are mainly to devote yourselves has been defined to be "the art or science of curing disease." Of two young men who commence life with equal capacities, and with other supposed equal conditions, it may be safely affirmed that success shall more abundantly crown him who enters upon his profession and pursues it from the motive of a love for that profession—not merely for the purpose of making money out of it, nor by means of it to weave garlands of fame to bind his brow, but because he loves it for its own sake. Love is but a little word of four letters, and yet it is a big word. It is the cohesive power that holds the world together; nay more, it is the grand principle which, proceeding from the throne of the Eternal, binds into harmony the system of worlds in the universe. It is the ,connecting link between man the creature, and God the creator.

Love of your profession will sweeten that labor, and make pleasant that toil by which alone you may expect to attain marked success. It is the intention that enables the dull and plodding to achieve results that genius can never hope for without it. It is love for the work in hand they have to do, that is stimulating and drawing forward men of science all over the world by constant toil and earnest thought, to add to the stock of knowledge and civilization. Then, gentlemen, go not forth to the discharge of your duties like a lazy, reluctant school-boy, driven to his book by the lash, but go forth heartily, cheerily and delightedly. He who constantly dreams

> "Of cutting foreign throats—
> Of breaches, ambuscades, Spanish blades,"

is fitted for the life of a soldier. So, let your profession be your thought by day and your dream by night; let it be, so to speak, mistress and sweetheart to you; let "the castles in air" you build be filled with native contributions that you are to bring there and place upon the altar of human progress and happiness.

Certainly the profession you have chosen is such as to stimulate the ambition of the noblest minds. What similar pursuit can compare with it in dignity and importance? Life is but a burden without the boon of health. At any moment we may need the services and the skill of the physician. He it is who helps us to land safely at "Port Natal;" he it is who attends us all along the voyage of life, and when its "fitful fever is o'er," he it is who smoothes our passage to the tomb.

The stoutest hearts, when disease overtakes them, are ready to dispatch a messenger in haste for the "dear doctor." Be prepared; gentlemen, to respond to the call with skill and alacrity. As long as happiness and health are so closely allied, it is impossible to overestimate your calling, or to set limits to the sphere of your usefulness. From the ninth to the thirteenth centuries of the Christian era, the persecuted Jews were the sole repositories of what little medical knowledge Europe possessed; and, hence, despite the laws which forbade them to administer remedies to Christians, we are told that they obtained access to the homes of the wealthy, to the courts of Sovereigns, and even to the palace of the Roman Pontiff.

Gentlemen, how can you fail to be in love with your profession, when you reflect upon the nature of the objects upon which you are to exercise it; when its duties are constantly enabling you to perceive "what a piece of work is man; how noble in reason, how infinite in faculties; in form and moving how expressive and admirable; in action how like an angel; in apprehension how like a God; the vanity of the world, the paragon of innocence?" You find him wasted by disease; your skill restores him to health and vigor. It is you who lead him by the hand from his sick room, to enjoy the glorious light and heat of the sun to breathe the balmy air of Heaven, and to go on his way rejoicing, like gladsome birds, that the rosy hue of health has come again to his cheek, and strength to his limbs.

Wherever the cry of distress is heard from disease, there is to be your place. Your mission is "to open the eyes of the blind, to unstop the deaf ears," and to say to the bedridden, "Arise, talk up thy bed and walk." With reverence I say, your vocation partakes more of the godlike than the merely human character. Love, then, your calling—love your fellow-man; then will your lives be grand successes.

Disease in its loathesome forms; abject misery in its degraded condition, might excite disgust in the refined sensibilities of your natures, but this talisman, love, worn in your hearts, will cause you to forget yourselves and to be interested only in the duty of relieving pain and suffering. It will enable you to endure the "winter's cold, the summer's heat," and to keep sleepless vigils by the bedside of the lowly and poverty-stricken. It will give you the soft tread and low and gentle tone of voice in the sick room, and the ear quick to catch the faintest symptom of a change in the patient's condition.

Disease in all its forms—pain and suffering—are your enemies. Combat with them, exterminate them, if possible; but their subjects, the human beings upon which they prey, are your friends. Love and cherish them as with a mother's tender care. Let their dependent state, and their implicit faith in your skill, be incentives to prove yourselves worthy the exalted trust. Be assured, gentlemen, you shall not be without a reward for your well-doing. The consciousness that you have been the means of making the sick well, shall be a source of happiness. There will be grateful hearts all around you to call you blessed.

Reputation, fame, glory and wealth lie scattered in the path before you. These are the prizes for which you are to contend; and fidelity, zeal, intelligence and patience are the means by which you are to grasp them.

The science of medicine is a vast field, which has been partially explored; there are zealous workers in that field all over the world. Be you, gentlemen, of that number. Whilst diligently attending to the routine of your daily duties, never forget that the practitioner of medicine owes a debt to the cause of science—to the world at large. Grand truths may be eliminated from observations made in single cases. If there is any science in your calling, it is only because disease has its laws, and that it may be counteracted and prevented by bringing to bear on it other laws that are superior to it. Be diligent in ascertaining what those laws are, so that you may benefit not only those who are in your narrow sphere, but also those whose faces you may never see—all mankind; and thus, gentlemen, will you make glad, also, hearts that beat in climes other than your own.

Gentlemen, may you have many patients; may none of them ever die, but may they all live to reward you with grateful hearts, and what is, perhaps, better, fat fees for your skill and fidelity.

